# Dynamic changes of HBV markers and HBV DNA load in infants born to HBsAg(+) mothers: can positivity of HBsAg or HBV DNA at birth be an indicator for HBV infection of infants?

**DOI:** 10.1186/1471-2334-13-524

**Published:** 2013-11-06

**Authors:** Tianyan Chen, Jing Wang, Yuling Feng, Zhi Yan, Tieying Zhang, Minghui Liu, Yun Bai, Hongxia Song, Hongli Liu, Yuan Yang, Jinfeng Liu, Yingli He, Yunru Chen, Shulin Zhang, Guihua Zhuang, Xiaofeng Liang, Zongyin Liu, Xiaguang Xu, Wei Chen, Yong Liu, Yingren Zhao

**Affiliations:** 1 School of Medicine, Xi’an Jiaotong University, Xi’an 710061, Shaanxi Province, China; 2 Xi’an Hangtian Hospital, Xi’an, Shaanxi, China; 3 Shangluo Central Hospital, Shangluo, Shaanxi, China; 4 Chinese Center for Disease Control and Prevention, Beijing, China; 5 Baoji Maternal and Child Care Service Center, Baoji, Shaanxi, China; 6 Ankang Central Hospital, Ankang, Shaanxi, China

## Abstract

**Background:**

Neither HBV DNA nor HBsAg positivity at birth is an accurate marker for HBV infection of infants. No data is available for continuous changes of HBV markers in newborns to HBsAg(+) mothers. This prospective, multi-centers study aims at observing the dynamic changes of HBV markers and exploring an early diagnostic marker for mother-infant infection.

**Methods:**

One hundred forty-eight HBsAg(+) mothers and their newborns were enrolled after mothers signed the informed consent forms. Those infants were received combination immunoprophylaxis (hepatitis B immunoglobulin [HBIG] and hepatitis B vaccine) at birth, and then followed up to 12 months. Venous blood of the infants (0, 1, 7, and 12 months of age) was collected to test for HBV DNA and HBV markers.

**Results:**

Of the 148 infants enrolled in our study, 41 and 24 infants were detected as HBsAg(+) and HBV DNA(+) at birth, respectively. Nine were diagnosed with HBV infection after 7 mo follow-up. Dynamic observation of the HBV markers showed that HBV DNA and HBsAg decreased gradually and eventually sero-converted to negativity in the non-infected infants, whereas in the infected infants, HBV DNA and HBsAg were persistently positive, or higher at the end of follow-up. At 1 mo, the infants with anti-HBs(+), despite positivity for HBsAg or HBV DNA at birth, were resolved after 12 mo follow-up, whereas all the nine infants with anti-HBs(−) were diagnosed with HBV infection. Anti-HBs(−) at 1 mo showed a higher positive likelihood ratio for HBV mother-infant infection than HBV DNA and/or HBsAg at birth.

**Conclusions:**

Negativity for anti-HBs at 1 mo can be considered as a sensitive and early diagnostic indictor for HBV infection in the infants with positive HBV DNA and HBsAg at birth, especially for those infants with low levels of HBV DNA load and HBsAg titer.

## Background

With the hepatitis B vaccination program implementation in China, hepatitis B surface antigen (HBsAg) carrier rate reduced from 9.75% in 1992 to 7.18% in 2006 [1]. While considering the large population of China, there are still mounts of newborns of HBsAg(+) mothers at high risk for hepatitis B virus (HBV) infection. Moreover, HBV infection of newborns is likely to cause chronic disease and serious subsequent complications.

Although combined immunoprophylaxis provides a high protective efficacy, it does not completely eradicate HBV transmission. HBV intrauterine infection is one of the main reasons for the failure of combined immunoprophylaxis, which represents 5%–10% of infants’ infection born to HBsAg(+) mothers [2-5].

Mother-to-infant transmission of HBV remains to be intensively studied. Currently, there is still no recognized diagnostic standard for HBV infection of infants. Early studies recommended that HBV DNA positivity in the cord blood can be used as a criterion for HBV mother-infant infection; however, the cord blood can be easily contaminated by the maternal blood. Zhu and Zhuang et al. provided evidences that testing of venous blood for HBsAg or HBV DNA of infants at birth was more accurate than cord blood for diagnosis of HBV infection [4,6]. Controversial data showed that about 10%-23% of infants from HBsAg(+) mothers with combined immunoprophylaxis displayed HBV DNA(+) or HBsAg(+) at birth, the positive rate gradually reduced during follow-up [4,7], therefore Zhu et al. proposed that infants whose HBV DNA or HBsAg remained positive for more than 3 months can be identified as having been infected [4]. Recently, new evidences recommended that infants who were seropositive for HBsAg and HBV DNA at 7 months could be identified as having acquired HBV infection [6-9]. Those data deepened our understanding of HBV infection of infants, development of a sensitive and early diagnostic indictor is needed for HBV infection of infants.

Other than positive rate, HBV markers titer and HBV DNA load also changes with ages. For the reason of placenta transmission, HBsAg was detected positive in infants even the non-infected ones at birth [8]. Jiang et al. showed that HBV DNA load, hepatitis B e antigen (HBeAg) and HBsAg titers at 12 months in HBV infected infants significantly increased as compared to birth [8]. According to our knowledge, no data reported the changes of HBsAg titer and HBV DNA load in those non-infected infants and the comparison of the HBV markers modes and quantification between infected and non-infected infants. From the aspect of quantification of HBV markers assay, most lower limit of HBV DNA detection as shown in the literatures was about 500 IU/ml or higher [2,7]. However, infants with HBV DNA below 500 IU/ml at birth, which detected negative with traditional detection system, were also at risk of infection [10]. With the development of more sensitive detection system, HBV DNA detection lower limit can be as low as 12 IU/ml as used in this study.

In this prospective, multi-centers study, kinetics of viral markers titer and HBV DNA load were investigated with more accurate assay methods, in infants treated with combined immunoprophylaxis by follow-up as long as 12 months, HBV markers modes and quantification were also compared, try to identify a sensitive and early indicator for HBV infection of infants.

## Methods

### Subjects

From November 2009 to August 2011, 148 pregnant women who were screened HBsAg positive during their prenatal care were recruited from 16 hospitals in Shaanxi Province, China. The inclusion criteria for participants were as follows: 20–40 years old, positivity for serum HBsAg, negative for anti-HBs. All participants in this study signed written informed consent forms for the participation of their infants upon birth. The study protocol was approved by the ethics committee of the First Affiliated Hospital of Xi’an Jiaotong University. Data files were kept locked to ensure confidentiality of respondents.

### Data and samples collection

After childbirth, data were collected from the recruited women’s medical records, which include fully medical care information before and after delivery.

Maternal venous blood was collected before delivery and neonatal venous blood was taken prior to combined immunoprophylaxis. The infants were consecutively followed up at 0, 1, 7, and 12 months of age (blood was taken before hepatitis B vaccine injection). Serum was separated for HBV DNA load tests and measurement of HBV serum markers titer (HBsAg, anti-HBs, HBeAg, and hepatitis B core antibody [anti-HBc]).

### Immunization schedule

Combined immunoprophylaxis scheduled as following, HBIG (200 IU; Hualan Biological Engineering Inc., Henan, China) and the first dose of the hepatitis B vaccine (Shenzhen Kangtai Biological Products Co. Ltd., Guangdong, China) at different injection sites within 12 h postpartum, followed by the other 2 doses of the hepatitis B vaccine at the ages of 1 and 6 months, respectively.

### Laboratory methods

HBV serum markers (HBsAg, anti-HBs, HBeAg, and anti-HBc) were quantified by the Abbott ARCHITECT HBsAg, anti-HBs, HBeAg, and anti-HBc assays, respectively (lower limits of the dynamic range: 0.05 IU/mL, 10.00 mIU/mL, 1.00 s/co, and 1.00 s/co, respectively; Abbott Laboratories, Chicago, USA). The HBV DNA load was measured by a real-time PCR-based Roche COBAS AmpliPrep/COBAS TaqMan HBV test (lower limit of the dynamic range: 12 IU/mL; Roche Molecular Systems). Liver function tests were performed with an automated bioanalyzer (Olympus AU5400, Japan). Genotypes were determined by an in-house nested PCR assay, as described in the previous study [11].

### Statistical analysis

EpiData 3.0 was used to establish the clinical database. Data were double-checked for transcription errors, and were then analyzed statistically with SPSS 13.0. Descriptive analysis (calculations of averages, frequencies, proportions, or rates) was conducted. The chi-square for R × C table test was used for comparison between more than 2 groups. Mixed ANOVA was used to compare trends and changes between 2 groups. Quantitative data were analyzed with Shapiro-Wilk test and Levene statistic for normality and homogeneity of variance, respectively. According to situation, correlations of two quantitative groups were analyzed with Pearson or Spearman correlation test. The positive likelihood ratio was used for assessing the value of a diagnostic indicator and calculated as: positive likelihood ratio = sensitivity/(1-specificity).

## Results

### Profiles of mothers and newborns

Among the 148 HBsAg(+) mothers enrolled, 41 (27.7%) were HBeAg(+), 38 (25.7%) had high levels of HBV DNA load (more than 10^5^ IU/mL), 129 (87.2%) showed a normal alanine aminotransferase (ALT) level at delivery, and 139 (93.9%) were infected with HBV genotype C (Table 1).

**Table 1:** Characteristics of mothers and infants enrolled in the study

**Mothers (Total mothers 148)**	
Age, years, Mean ± SD (range)	25.9 ± 3.8 (20–38)
Elevated ALT levels, N (%)*	19 (12.8%)
ALT, Mean ± SD, (range), U/L	25.5 ± 30.1 (4.0–249.0)
AST, Mean ± SD, (range), U/L	30.6 ± 34.4 (11.1–343.0)
HBeAg(+), N (%)	41 (27.7%)
HBV DNA more than 10^5^ IU/mL, N (%)	38 (25.7%)
Genotype C, N (%)	139 (93.9%)
**Neonates (Total neonates 148)**	
Female: Male	70:78
HBsAg(+) at birth, N (%)	41 (27.7%)
HBeAg(+) at birth, N (%)	35 (23.6%)
HBV DNA(+) at birth, N (%)	24 (16.2%)

* Elevated ALT level was defined as the value of ALT level is upper 40 U/L.

Among 148 infants, 70 infants (47.3%) were female, 78 (52.7%) were male. The positive rates of HBV DNA, HBsAg, HBeAg, and anti-HBc in the newborns at birth were 16.2%, 27.7%, 23.6%, and 100%, respectively. All newborns were negative for anti-HBs (i.e., anti-HBs titer was less than 10 mIU/mL) at birth (Table 1).

### Correlation of HBsAg and HBV DNA between mothers and newborns

**Figure 1:**
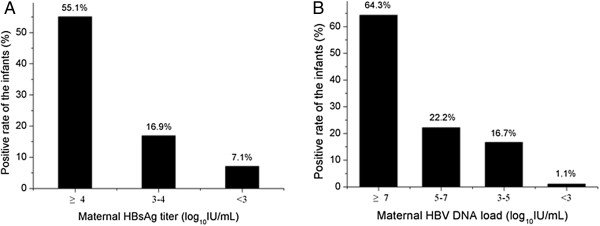
**Correlation of HBsAg and HBV DNA between mothers and newborns.** HBsAg(+) and HBV DNA(+) rates of the infants at birth in different levels of maternal HBsAg titer **(A)** and HBV DNA load **(B)** groups.

Of the 148 infants born to HBsAg(+) mothers, 41 were detected HBsAg positive at birth. To discover the relationship of HBsAg between mothers and infants, HBsAg titer of those 41 mother-infant pairs were analyzed, no correlation was found between the pairs (r = 0.188, *p* = 0.239). However, when the mothers were stratified into high, intermediate and low levels of HBsAg titer, as shown in Figure 1A, infants from high HBsAg level group were at higher risk of HBsAg positive rate at birth (*p* < 0.001).

Attributed to a sensitive HBV DNA detection system, 24 infants were detected positive (> 12 IU/mL), which was much higher than data reported by literature [8,10]. Similar with HBsAg, no correlation was found between the double HBV DNA positive mother-infant pairs (r = 0.370, *p* = 0.076). Further analysis of stratified mothers with HBV DNA level showed similarity to HBsAg, as indicated in Figure 1B, high level HBV DNA mother-groups confer greater risk to infants HBV DNA positive at birth (*p* < 0.001).

### Positive rate of HBV markers and HBV DNA in infants

**Figure 2:**
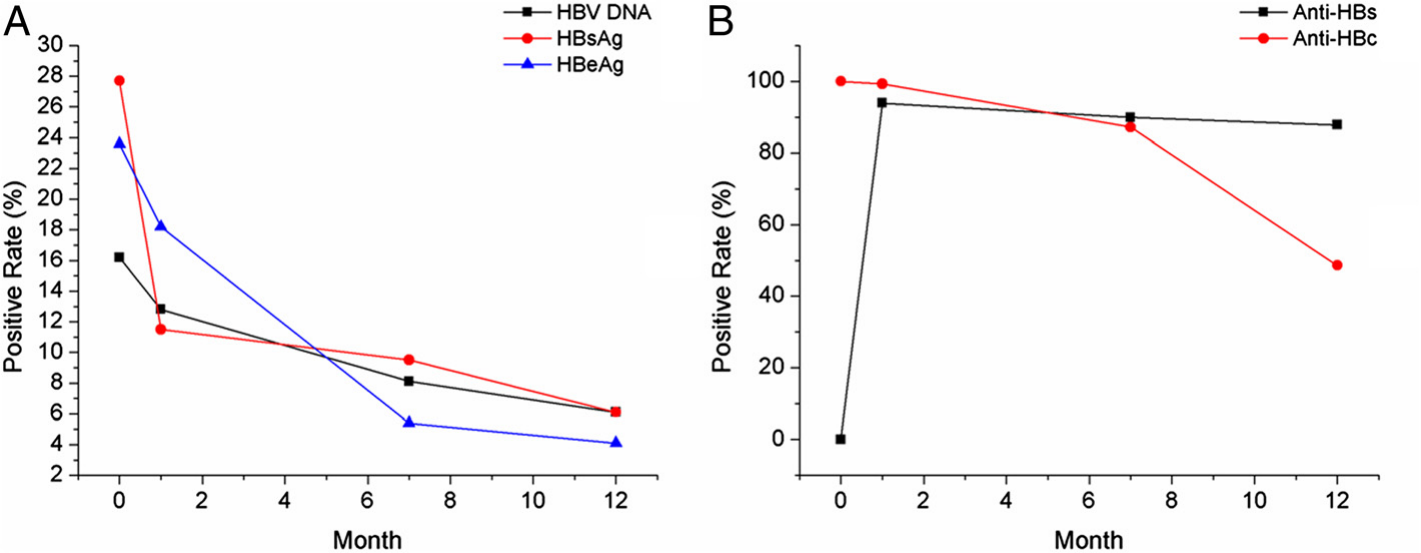
**HBV DNA(+), HBsAg(+), HBeAg(+), anti-HBc(+), and anti-HBs(+) rates in infants.** Positive rates of **(A)** HBV DNA, HBsAg, and HBeAg and **(B)** anti-HBc and anti-HBs at birth, 1 mo, 7 mo and 12 mo.

Next, we investigated changes in the positive rates of HBV markers and HBV DNA over the first year of the infants’ lives. The rates of HBV DNA(+), HBsAg(+), HBeAg(+), and anti-HBc(+) in infants reduced gradually during the follow-up (*χ*^2^: 9.67, 592.01, 36.83, and 190.7, respectively; P = 0.022, <0.001, <0.001, and <0.001, respectively). Although anti-HBs is a placenta- transmittable antibody, because all the mothers enrolled were anti-HBs negative, it was not surprising that all the infants were negative for anti-HBs at birth. Attributed to the combined immunoprophylaxis, 93.9% (139/148), 89.9% (133/148) and 87.8% (130/148) of the infants were detected protective anti-HBs at 1 month, 7 months and 12 months, respectively (Figure 2). Nine (6.1%) infants who were detected anti-HBs(−) at 1 month were anti-HBs(−) and HBV DNA(+) at both 7 months and 12 months. It was the nine infants that were diagnosed with HBV infection by follow-up. The failure rate of combined immunoprophylaxis in this study was comparable with literature (5%-10% *vs.* 6.1%) [2-5].

Among mothers of the 9 infected infants, 6 of them had high HBV DNA load (>10^5^ IU/mL), and all of them had high levels of HBsAg (>10^4^ IU/mL). Six mothers were HBeAg(+), and 3 were HBeAg(−) (Table 2). All 9 mothers were positive for HBV genotype C, the dominant genotype in China as shown in our previous study [12].

**Table 2:** Characteristics of the 9 mother–infant pairs

	**Mothers**			**Infants**									
**Patient**	**HBsAg**	**HBeAg**	**HBV DNA**	**HBsAg at birth**	**HBeAg at birth**	**HBV DNA at birth**	**HBsAg at 1 month**	**HBeAg at 1 month**	**HBV DNA at 1 month**	**Anti-HBs at 1 month**	**HBsAg at 7 month**	**HBeAg at 7 month**	**HBV DNA at 7 month**
**No.**	**(IU/ml)**	**(s/co)**	**(log**_**10**_**IU/ml)**	**(IU/ml)**	**(s/co)**	**(log**_**10 **_**IU/ml)**	**(IU/ml)**	**(s/co)**	**(log**_**10 **_**IU/ml)**	**(mIU/ml)**	**(IU/ml)**	**(s/co)**	**(log**_**10 **_**IU/ml)**
1	12462.2	1488.8	6.44	0.10	5.20	1.72	0.07	3.03	1.45	2.94	12340.14	894.68	6.72
2	>125000*	1969.5	8.34	9.93	20.44	8.12	>125000	1275.80	8.41	0	>125000	1516.68	8.53
3	83887.1	1540.9	7.49	9210.06	644.43	8.02	>125000	1236.10	8.08	0	>125000	1463.10	8.16
4	>125000	1898.8	7.03	0.08	19.95	7.68	0.08	3.25	8.54	0.02	>125000	1089.89	8.55
5	11359.4	0.6	4.64	1321.33	0.57	3.07	>125000	89.47	4.66	0	>125000	309.74	8.80
6	24560.1	1017.9	9.26	>125000	976.43	5.86	>125000	987.33	7.89	0	>125000	1167.23	8.90
7	19740.3	0.5	4.73	0.07	0.46	3.41	1675.44	0.72	4.53	0	1743.25	0.35	4.64
8	12049.5	0.7	3.43	0.72	0.57	1.95	2345.63	0.56	3.14	0.17	3142.317	0.59	3.63
9	>125000	1241.1	8.47	>125000	130.76	5.65	>125000	123.57	8.52	0.08	>125000*	790.13	8.56

>125000: The HBsAg titer exceeded the upper limit.

### Comparison of the dynamic changes in HBV markers titer and HBV DNA load between infected and uninfected infants

**Figure 3:**
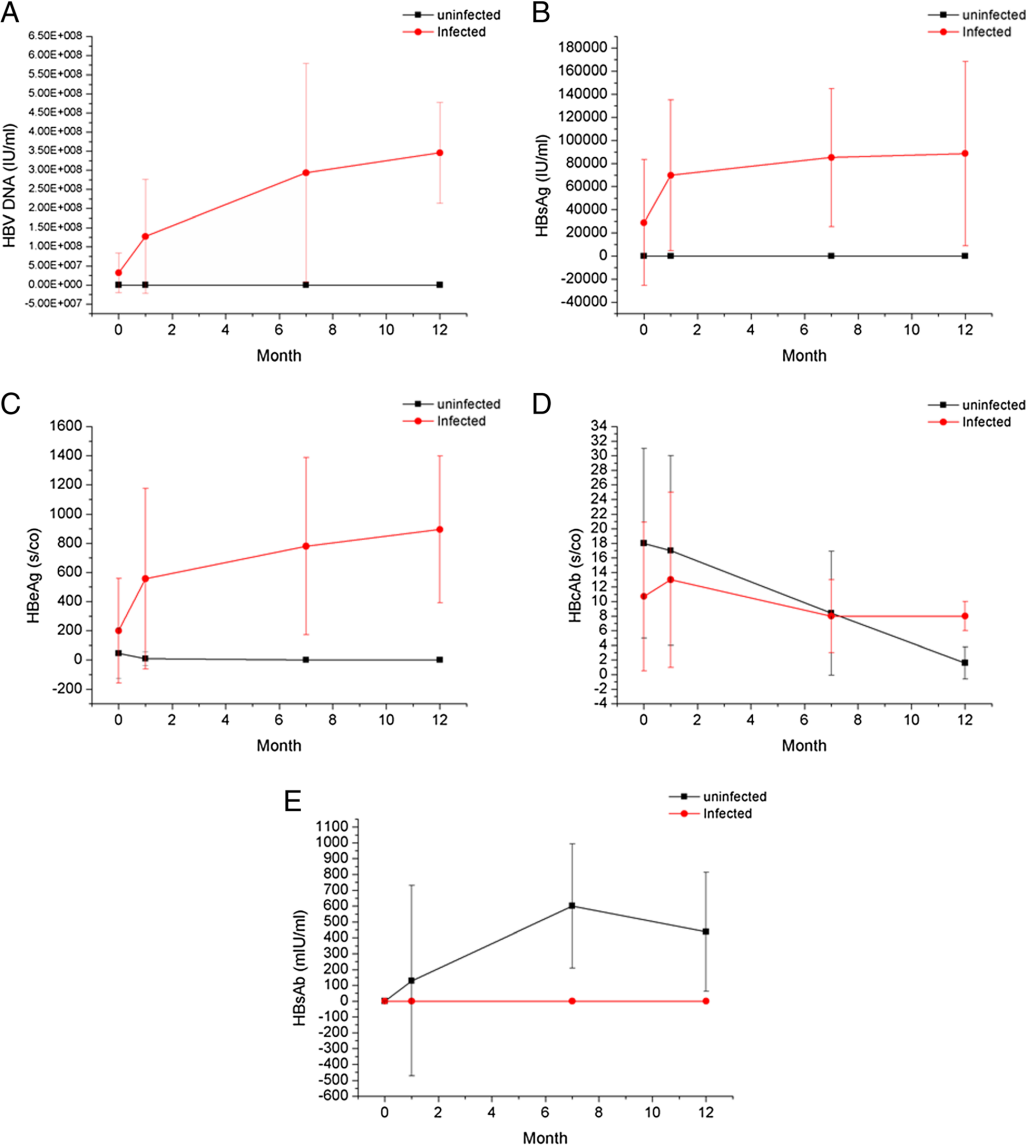
**Dynamic changes in HBV surface markers titer and HBV DNA load in infected and uninfected infants.** Dynamic changes in HBV DNA **(A)**, HBsAg **(B)**, HBeAg **(C)**, anti-HBc **(D)**, and anti-HBs **(E)** in infected and uninfected infants were compared at birth, 1 mo, 7 mo and 12 mo.

As showed before, 9 infants with continuous HBV DNA(+) and HBsAg(+) were diagnosed with HBV infection. Dynamic tendency in HBV DNA load, HBsAg, HBeAg, anti-HBc, and anti-HBs titers were significantly different between 9 infected infants and 139 uninfected infants (F = 2.13 × 10^10^, *P* < 0.001; F = 87.78, *P* < 0.001; F = 2.59 × 10^7^, *P* < 0.001; F = 6.73, *P* < 0.001; and F = 2.82, *P* = 0.047, respectively; Figure 3). HBV DNA load, HBsAg and HBeAg titers of the 9 HBV infected infants increased gradually, while 139 uninfected infants went opposite way. Nine infected infants presented anti-HBs(−) even under consecutive detection, whereas the titers in the 139 non-infected infants increased.

**Figure 4:**
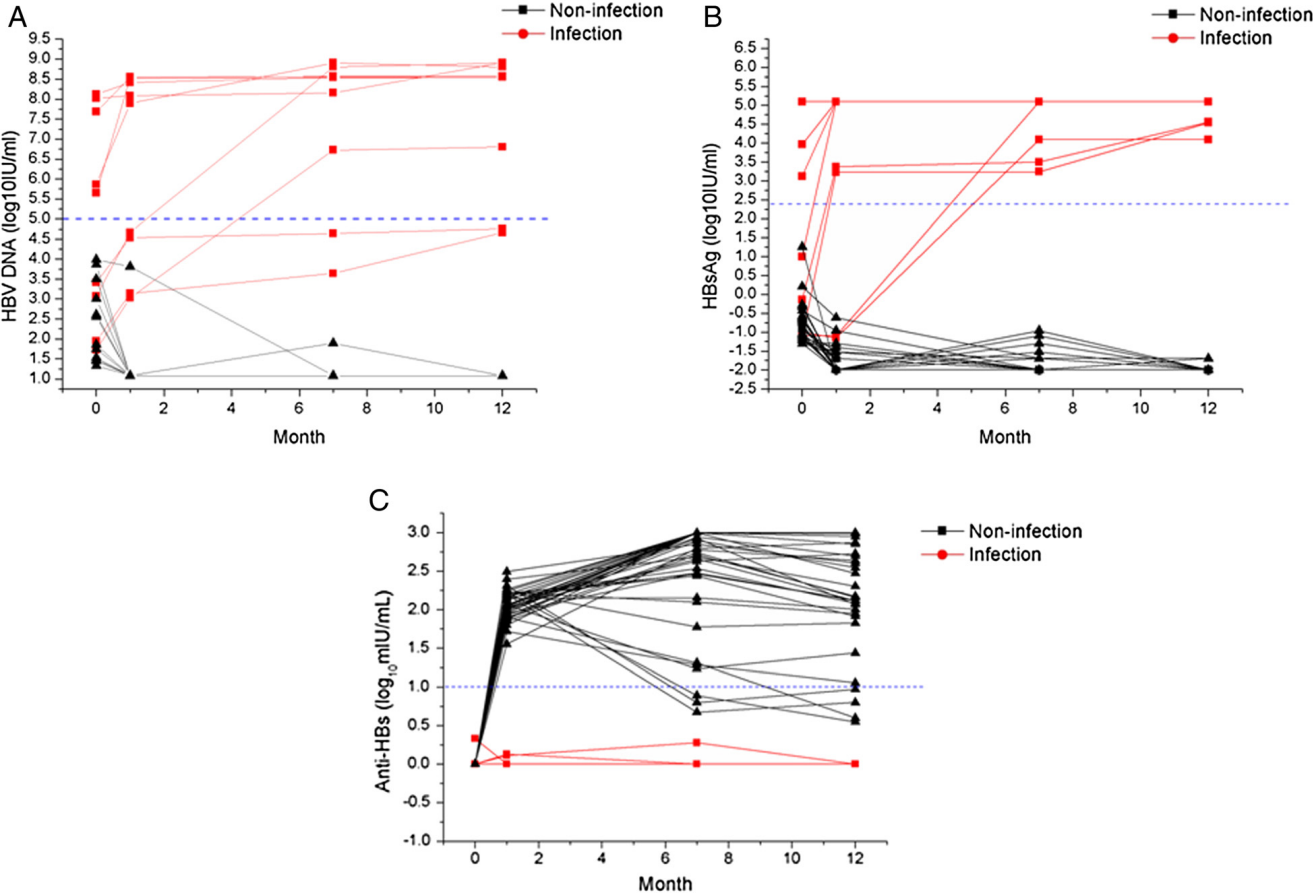
**Dynamic changes in HBV DNA load, HBsAg titer and anti-HBs titer in infants who were HBsAg(+) and HBV DNA(+) at birth.** Dynamic changes in HBV DNA load **(A)**, HBsAg titer **(B)** and anti-HBs titer **(C)** in infants with HBsAg(+) and/or HBV DNA(+) at birth were compared at birth, 1 mo, 7 mo and 12 mo.

At birth, 24 newborns were positive for HBV DNA, 41 newborns were positive for HBsAg. 17 newborns were double positive for HBV DNA and HBsAg. Although evidences exists that HBV DNA and HBsAg can be diagnostic indictors for HBV infection of infants, the specificity and sensitivity of those two markers remains to be studied. Ours results showed that high levels of HBV DNA load at birth (more than 10^5^ IU/mL) detected in 5 infants was a robust predictive marker for HBV infection at 12 months. Among those 19 infants with low levels of HBV DNA (less than 10^5^ IU/mL), 15 infants who were anti-HBs detected positive at 1 month were negative for HBV DNA and HBsAg at the age of 12 months, 4 infants were persistently positive for HBV DNA and HBsAg and negative for anti-HBs (Figure 4A).

As we mentioned before, conventional quantification assay detects HBV DNA lower limit at about 500 IU/ml, however, as shown in Figure 4, 2 infants with HBV DNA load less than 500 IU/ml at birth were diagnosed with HBV infection at 12 months, which call for a sensitive HBV DNA detection system. In this study, the lower limit of HBV DNA detection was as low as 12 IU/ml, 100% HBV infected infants at 12 months were detected HBV DNA positive at birth.

As shown in Figure 4, 4 infants with high levels of HBsAg titer (more than 250 IU/mL) at birth, persistently positive for HBV DNA and HBsAg, were diagnosed with HBV infection; 32 in 37 infants with low levels of HBsAg titer (from 0.05 IU/mL to 250 IU/mL) eliminated HBV virus at the age of 12 months, other 5 infants with persistently HBV DNA(+) and HBsAg(+), anti-HBs was negative.

### Anti-HBs(+) at 1 mo is an early indictor of HBV infection

Since neither HBV DNA(+) nor HBsAg(+) at birth can be an indictor of HBV infection, a more sensitive and specific indicator is needed for early predicting HBV infection. Ours data demonstrated that all infants were diagnosed with HBV infection at 12 mo were anti-HBs(−) at the 1 month (anti-HBs titers were from 0 mIU/mL to 2.94 mIU/mL). More important, 100% infants with anti-HBs(+) at 1 mo were free of HBV infection at 12 mo (Figure 4C), thus anti-HBs(+) at 1mo may be a reliable early indictor for HBV infection. Further analysis showed that the positive likelihood ratio of anti-HBs(−) 1mo was much higher than HBV DNA(+) and/or HBsAg(+) (Table 3), suggesting a good indictor for early HBV infection.

**Table 3:** Positive likelihood ratio of diagnostic indicators for chronic HBV-infected infants

**Diagnostic indicators**	**N**	**Infected**		**Uninfected**		**Positive likelihood ratio**
		**N**	**True positive ratio**	**N**	**False positive ratio**	
HBV DNA(+) at birth	21	9	9/9 = 1	12	12/139 = 0.086	11.6
HBsAg(+) at birth	41	9	9/9 = 1	32	32/139 = 0.230	4.34
HBV DNA- and HBsAg- positive at birth	18	9	9/9 = 1	9	9/139 = 0.065	15.4
Anti-HBs(−) at 1 month old	9	9	9/9 = 1	0	0/139 = 0	+∞

## Discussion

The reported rates of HBV mother-infant infection in China ranged from 3.2% to 40.1% [6-10,13,14]. Following reasons were thought attributed to this significant variation: 1) the sample sizes of some studies were not large enough, 2) since HBV DNA markers in newborns change during the first year, follow-up should be last to stable phase, and 3) the methods detecting HBV markers were not sensitive enough. In current prospective, multi-centers study, mothers screen HBsAg(+) and their newborns were enrolled and followed-up for as long as 12 months. More sensitive and specific tests were employed to detect HBV DNA load and HBV markers titer.

At birth, a substantial proportion of infants were detected HBV markers positive, following 12 months follow-up, we found that the positive rates of HBV DNA and HBsAg gradually reduced. The rate of infants with HBsAg(+) or HBV DNA(+) at birth resolved after 12 months follow-up was as high as 81.3%, suggesting serum HBV DNA(+) and HBsAg(+) at birth can not serve as the diagnostic markers for HBV infection. Although HBV DNA or HBsAg is a widely used diagnostic marker for HBV infection in adults, for infants, both HBV DNA [14-16] and HBsAg [16] may cross the human placenta via partial placental leakage or via the “cellular route”. In current study, all the newborns were from HBV infected mother, which ensured feasibility for infants to passively acquire HBV markers from mothers. Secondly, if those markers detected in infants passively pass form their mothers, infants from highly replication mothers were more likely positive for those markers. Supported by ours data, the infants born to mothers with high levels of HBV DNA load or high level of HBsAg were at greater risk of positivity for HBV DNA or HBsAg at birth, when compared with those infants of mothers with low level HBV DNA and HBsAg. Thirdly, since those markers passively came from their mothers, but not actively from HBV replication in infants’ liver, all those markers were fated to disappear eventually. In our observation, HBsAg titer and HBV DNA load decreased gradually and eventually sero-converted to negativity in the non-infected infants. On the contrary, all infected infants presented persistently HBV markers positive. All the infants with high HBV DNA load (>10^5^ IU/mL) and/or HBsAg titer (>250 IU/mL) at birth identified HBV infection eventually. Although most of infants with low levels of HBV DNA load (<10^5^ IU/mL) and/or HBsAg titer (<250 IU/mL) at birth were free of HBV at 12 months, a small proportion of infants were persistent for HBV DNA(+) and HBsAg(+) during the follow-up, which supported the notion that a sensitive detection methods, as employed in our study, should be used for HBV infection screening.

As shown above, both HBV DNA and HBsAg in infants may be from their mothers. A non-mother-origin marker is desperately needed for early diagnosis of HBV infection. Although anti-HBs also pass through placenta theoretically, all the mothers were anti-HBs(−). We propose that anti-HBs probably serve as such marker. Our data demonstrated that the infants with anti-HBs(+) at 1 month, despite positivity for HBsAg or HBV DNA at birth, were detected negative after 12 months follow-up, whereas all the infants with anti-HBs(−) at 1 months were identified as HBV infection. Additionally, the positive likelihood ratio of anti-HBs(−) at 1 month is the highest among all the markers analyzed. Thus, negativity for anti-HBs at 1 month can be considered as a sensitive and early diagnostic indictor for HBV infection in infant with HBV DNA(+) and HBsAg(+) at birth, especially for those infants with low levels of HBV DNA load and HBsAg titer.

Although revealed by this study that the anti-HBs(−) at 1 month is sensitive and early indicator for HBV infection, enlarged sample size is needed to confirm if this indicator could be a diagnostic marker which can be acceptable used in clinic care.

## Conclusions

This prospective, multi-centers study showed that the HBV-infection rate of infants born to HBsAg-positive mothers was 6.1% in Shaanxi Province, China. HBV DNA(+) and HBsAg(+) at birth can not serve as the diagnostic markers for HBV mother-infant infection. Negativity for anti-HBs at 1 month can be considered as a sensitive and early diagnostic indictor for HBV infection in infant with positive HBV DNA and HBsAg at birth, especially for those infants with low levels of HBV DNA load and HBsAg titer.

## Abbreviations

ALT: Alanine aminotransferase; AST: Aspartate aminotransferase; anti-HBc: Hepatitis B core antibody; anti-HBe: Hepatitis B e antibody; anti-HBs: Hepatitis B surface antibody; HBeAg: Hepatitis B e antigen; HBIG: Hepatitis B immune globulin; HBsAg: Hepatitis B surface antigen; HBV: Hepatitis B virus; PCR: Polymerase chain reaction.

## Competing interests

The authors declare that they have no competing interests.

## Authors’ contributions

TC and JW carried out the experiments and drafted the manuscript. YF, ZY, TZ, ML, YB, HS, HL, YY, JL, YH, YC, ZL, and XX collected the blood sample of the mothers and the infants and acquired the individual patient data. SZ, GZ, XL, and WC helped in designing the study. YZ and YLiu are the corresponding authors for this manuscript. They also conceived and coordinated the study. All authors read and approved the final manuscript.

## Pre-publication history

The pre-publication history for this paper can be accessed here:

http://www.biomedcentral.com/1471-2334/13/524/prepub
